# An Unsuspected Adult Intestinal Malrotation Presenting With Left-Sided Appendicitis, With the Diagnosis First Suggested by Purulent Drainage From the Appendiceal Orifice During an Elective Colonoscopy

**DOI:** 10.7759/cureus.116872

**Published:** 2026-09-24

**Authors:** Martim Rente, Arnaldo Machado, Ana Martins, Ines Matias, Manuel Cavalho

**Affiliations:** 1 General Surgery, Unidade de Saúde Local do Alentejo Central, Évora, PRT; 2 Surgery, Hospital do Espírito Santo de Évora, Évora, PRT; 3 General Surgery, Hospital do Espírito Santo de Évora, Évora, PRT

**Keywords:** adult intestinal malrotation, appendiceal orifice, colonoscopy complications, diagnosis of acute appendicitis, emergency laparoscopic appendectomy, left-sided acute appendicitis, colonoscopy

## Abstract

Acute appendicitis typically presents with right lower quadrant abdominal pain. Congenital abnormalities of intestinal rotation may alter the position of the cecum and appendix, resulting in atypical clinical manifestations and potentially delaying diagnosis. Intestinal malrotation is usually diagnosed during childhood but may remain asymptomatic and undetected until adulthood.

We report the case of a 63-year-old man who underwent elective colonoscopy following a positive fecal occult blood test. He reported abdominal pain beginning approximately 24 hours before the procedure, which he initially attributed to bowel preparation. During colonoscopy, purulent material was observed filling the appendiceal orifice, raising suspicion of acute appendicitis. The patient was referred to the emergency department, where examination revealed isolated tenderness of the left iliac fossa without right-sided abdominal pain. Laboratory evaluation showed no leukocytosis, 76% neutrophils, and an elevated C-reactive protein level of 6.7 mg/dL. Computed tomography demonstrated intestinal malrotation, with the colon predominantly located on the left side of the abdomen and pelvis, and an inflamed appendix arising from the abnormally positioned cecum to the left of the midline. Laparoscopic appendectomy was performed using right-sided working ports. Intraoperative findings demonstrated acute phlegmonous appendicitis without exudate, and histopathological examination confirmed acute phlegmonous appendicitis with focal serositis. The postoperative course was uncomplicated, and the patient was discharged on the first postoperative day.

This case illustrates an unusual presentation of acute appendicitis associated with previously undiagnosed intestinal malrotation, in which the initial diagnostic suspicion arose incidentally during colonoscopy. Recognition of anatomical variants and appropriate use of cross-sectional imaging are important when evaluating atypical presentations of common surgical emergencies.

## Introduction

Acute appendicitis is one of the most common causes of acute abdominal pain requiring emergency surgery. Although right lower quadrant pain represents its classical presentation, the location of symptoms may vary considerably according to the anatomical position of the appendix. Left-sided acute appendicitis is uncommon and is particularly associated with congenital anatomical abnormalities such as situs inversus totalis and intestinal malrotation [1].

Intestinal malrotation results from abnormal rotation and fixation of the midgut during embryological development. Although predominantly recognized during infancy and childhood, malrotation may remain clinically silent and be discovered incidentally during adulthood. A large computed tomography (CT)-based series of adults with intestinal malrotation demonstrated that a substantial proportion of patients were asymptomatic at diagnosis, highlighting how this congenital abnormality may remain unsuspected well into adult life [2].

Abnormal positioning of the cecum and appendix in intestinal malrotation can substantially alter the expected clinical presentation of acute appendicitis. A left-sided appendix may result in left lower quadrant pain and mimic more common conditions such as acute diverticulitis, renal colic, or colitis [1,3,4]. CT is particularly valuable in this setting because it can simultaneously identify appendiceal inflammation and characterize the underlying rotational abnormality [2-5].

Colonoscopy is not routinely used in the diagnosis of acute appendicitis. Nevertheless, inflammatory changes involving the appendiceal orifice, including edema, bulging, erythema, and purulent drainage, have occasionally led to the diagnosis of appendicitis during colonoscopy performed for other indications [6-8]. We report an unusual case of left-sided acute appendicitis in a 63-year-old man with previously undiagnosed intestinal malrotation, in whom the initial suspicion of appendicitis arose after purulent material was incidentally identified at the appendiceal orifice during elective colonoscopy.

## Case presentation

A 63-year-old man was referred to the emergency department following an elective colonoscopy performed after a positive fecal occult blood test. The patient reported abdominal pain of approximately 24 hours' duration before the colonoscopy. He had initially attributed the discomfort to the bowel preparation required for the procedure.

During colonoscopy, purulent material was observed filling the appendiceal orifice, raising suspicion of acute appendicitis (Figure 1). The patient was consequently referred directly to the emergency department for surgical assessment. His relevant medical history included arterial hypertension, gout, dyslipidemia, and allergic rhinitis. He had no previous surgical history and no known drug allergies.

**Figure 1:**
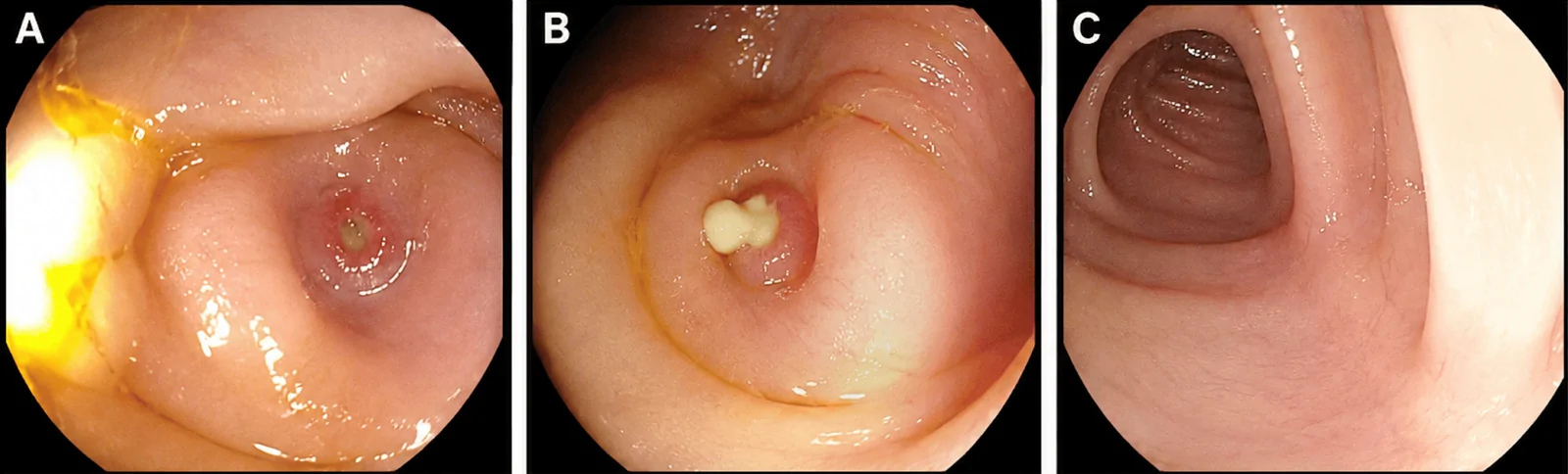
Colonoscopic appearance of the appendiceal orifice (A) Appendiceal orifice with surrounding mucosal erythema and inflammatory changes. (B) Purulent material draining from the appendiceal orifice, raising suspicion of acute appendicitis. (C) Additional colonoscopic view of the cecal region.

On examination, the patient was apyretic and hemodynamically stable. The abdomen was soft and non-distended, with tenderness in the left iliac fossa. There was no tenderness in the right iliac fossa or the remaining abdominal quadrants and no clinical evidence of generalized peritoneal irritation.

Laboratory investigations demonstrated no leukocytosis, with 76% neutrophils and an elevated C-reactive protein level of 6.7 mg/dL. The remaining laboratory findings were largely unremarkable and are summarized, together with the corresponding reference ranges, in Table 1. 

**Table 1:** Laboratory findings at presentation Laboratory values obtained during the patient's initial emergency department assessment. Reference ranges correspond to those provided by the institutional laboratory. INR, international normalized ratio.

Laboratory parameter	Result	Reference range
Red blood cell count	4.33 × 10⁶/µL	4.32-5.66 × 10⁶/µL
Hemoglobin	13.5 g/dL	13.3-16.7 g/dL
Hematocrit	40.7%	39.0%-50.0%
Mean corpuscular volume	94.1 fL	82.0-98.0 fL
Mean corpuscular hemoglobin	31.3 pg	27.3-32.6 pg
Mean corpuscular hemoglobin concentration	33.3 g/dL	31.6-34.9 g/dL
Red cell distribution width	14.3%	9.9%-15.5%
White blood cell count	8.5 × 10³/µL	3.7-9.5 × 10³/µL
Neutrophils	76.1%	47.2%-77.6%
Lymphocytes	15.9%	7.0%-36.0%
Monocytes	6.2%	4.0%-8.0%
Eosinophils	1.2%	2.0%-4.0%
Basophils	0.5%	0.0%-1.0%
Platelet count	317 × 10³/µL	170-430 × 10³/µL
Prothrombin time	11.80 s	9.90-12.80 s
INR	1.06	0.80-1.20
Glucose	104 mg/dL	60-95 mg/dL
Urea	28 mg/dL	19-43 mg/dL
Creatinine	0.94 mg/dL	0.66-1.25 mg/dL
Estimated glomerular filtration rate	85.59 mL/min/1.73 m²	>60 mL/min/1.73 m²
Sodium	139 mmol/L	137-145 mmol/L
Potassium	3.9 mmol/L	3.5-5.1 mmol/L
C-reactive protein	6.7 mg/dL	<1.0 mg/dL

Given the unusual left-sided abdominal pain and the colonoscopic finding suggestive of appendicitis, abdominal and pelvic CT was performed. CT demonstrated findings consistent with intestinal malrotation (Figure 2). The colon was predominantly located on the left side of the abdomen and pelvis.

**Figure 2:**
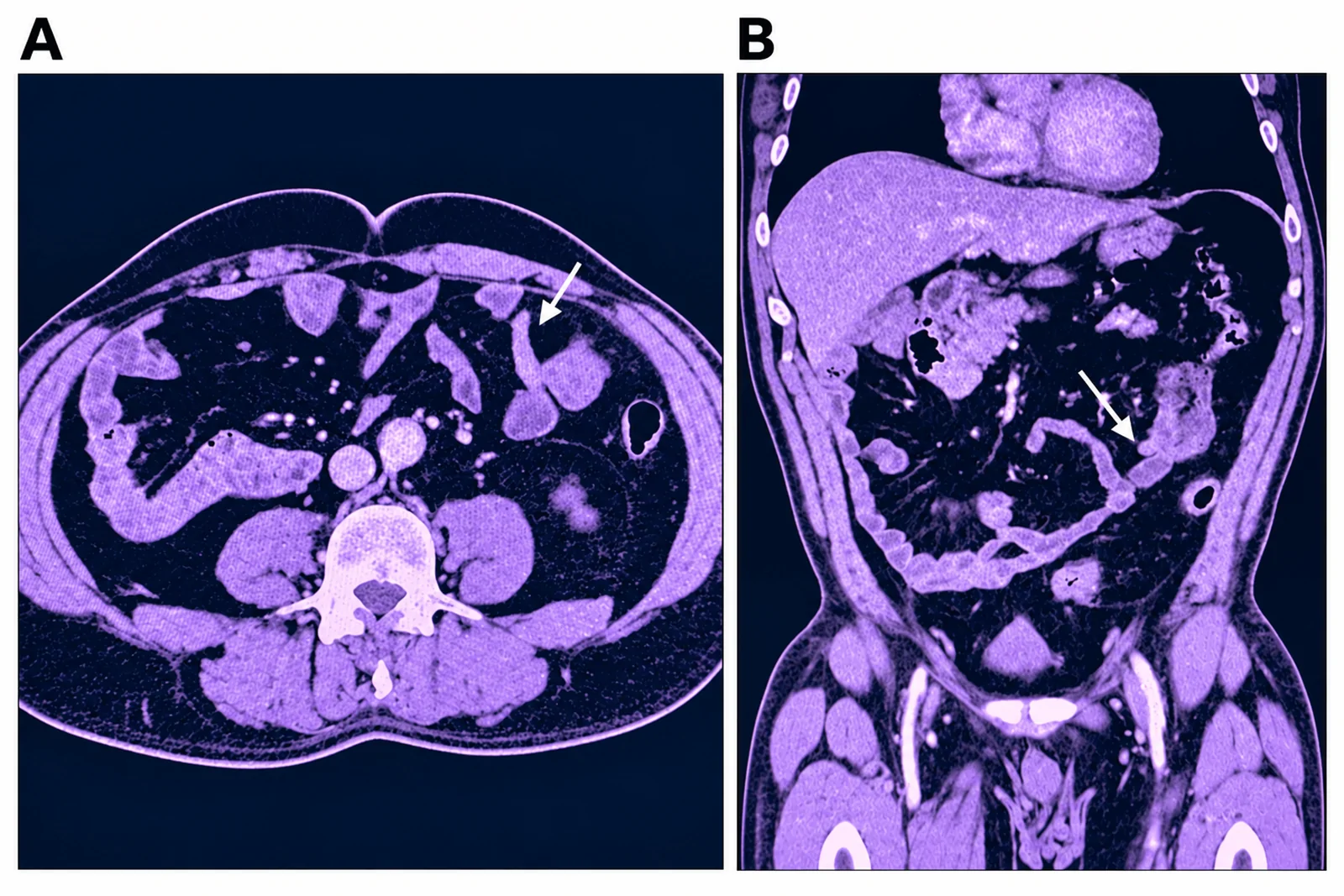
Computed tomography findings of acute appendicitis associated with intestinal malrotation A) Axial CT image demonstrating the inflamed appendix (arrow) located to the left of the midline, with a maximal proximal diameter of approximately 9 mm and mild surrounding inflammatory fat stranding. (B) Anteroposterior reconstructed computed tomography image demonstrating the abnormal bowel arrangement associated with intestinal malrotation, with the colon predominantly located on the left side of the abdomen.

A tubular structure arising from the cecum and compatible with the appendix was identified to the left of the midline in the lower abdomen, approximately at the level of the umbilicus. The appendix measured approximately 9 mm proximally and 6 mm distally and was associated with mild inflammatory changes in the surrounding fat, supporting the diagnosis of acute appendicitis. Small-bowel loops were of normal caliber, and no additional significant abnormalities were identified (Figure 2). The combined clinical, endoscopic, and radiological findings supported the diagnosis of acute appendicitis in the setting of previously undiagnosed intestinal malrotation.

Differential diagnosis

The principal diagnostic challenge was the presence of isolated left iliac fossa pain in the absence of right-sided abdominal tenderness. Given the patient's age and clinical presentation, acute diverticulitis was an important differential diagnosis. Left-sided renal colic and inflammatory or infectious colonic conditions were also considered. Appendicitis associated with intestinal malrotation can mimic these conditions because the inflamed appendix may be located entirely within the left abdomen [3,4].

Following identification of intestinal malrotation on CT, complications directly associated with malrotation, particularly intestinal obstruction and midgut volvulus, also required consideration. However, the small-bowel loops were of normal caliber, and there was no radiological evidence of obstruction or volvulus. The colonoscopic visualization of purulent material at the appendiceal orifice, together with the CT findings, established the diagnosis of acute appendicitis.

Treatment

The patient was admitted for urgent laparoscopic appendectomy after informed consent was obtained. Cefoxitin 2 g was administered intravenously approximately 30 minutes before surgery as antibiotic prophylaxis. The procedure was performed under general anesthesia with the patient in the supine position.

Pneumoperitoneum was established using an open technique at the umbilicus. Given the abnormal position of the cecum and appendix, the working ports were placed predominantly on the right side. A 10-mm right paraumbilical port, a 10-mm port in the right iliac fossa, and a 5-mm port in the right hypochondrium were used.

Laparoscopic exploration confirmed the abnormal intestinal arrangement and demonstrated acute phlegmonous appendicitis without exudate. The appendiceal artery was controlled using a polymer locking clip. The appendix was dissected to its base, which was secured with two polymer locking clips before division. The specimen was retrieved using a laparoscopic extraction bag.

The umbilical port was closed in layers, followed by skin closure with absorbable monofilament sutures and tissue adhesive. The operative duration was approximately 45 minutes, and no intraoperative complications occurred.

Outcome and follow-up

The postoperative course was uncomplicated. On the first postoperative day, the patient was clinically well, apyretic, and hemodynamically stable. He tolerated mobilization and an oral diet without nausea or other complications. Postoperative pain was mild and adequately controlled.

Physical examination demonstrated a soft abdomen with only mild discomfort on palpation of the left flank and no guarding. The surgical wounds showed satisfactory healing without evidence of early complications. The patient was discharged home on the first postoperative day.

Histopathological examination confirmed acute phlegmonous appendicitis with focal serositis (Figures 3, 4). At outpatient follow-up approximately one month after surgery, the patient remained asymptomatic, with a painless abdomen, appropriately healed surgical wounds, and no evidence of incisional hernia. He was subsequently discharged from surgical follow-up. Written informed consent for the use of clinical images and publication of the case was obtained from the patient.

**Figure 3:**
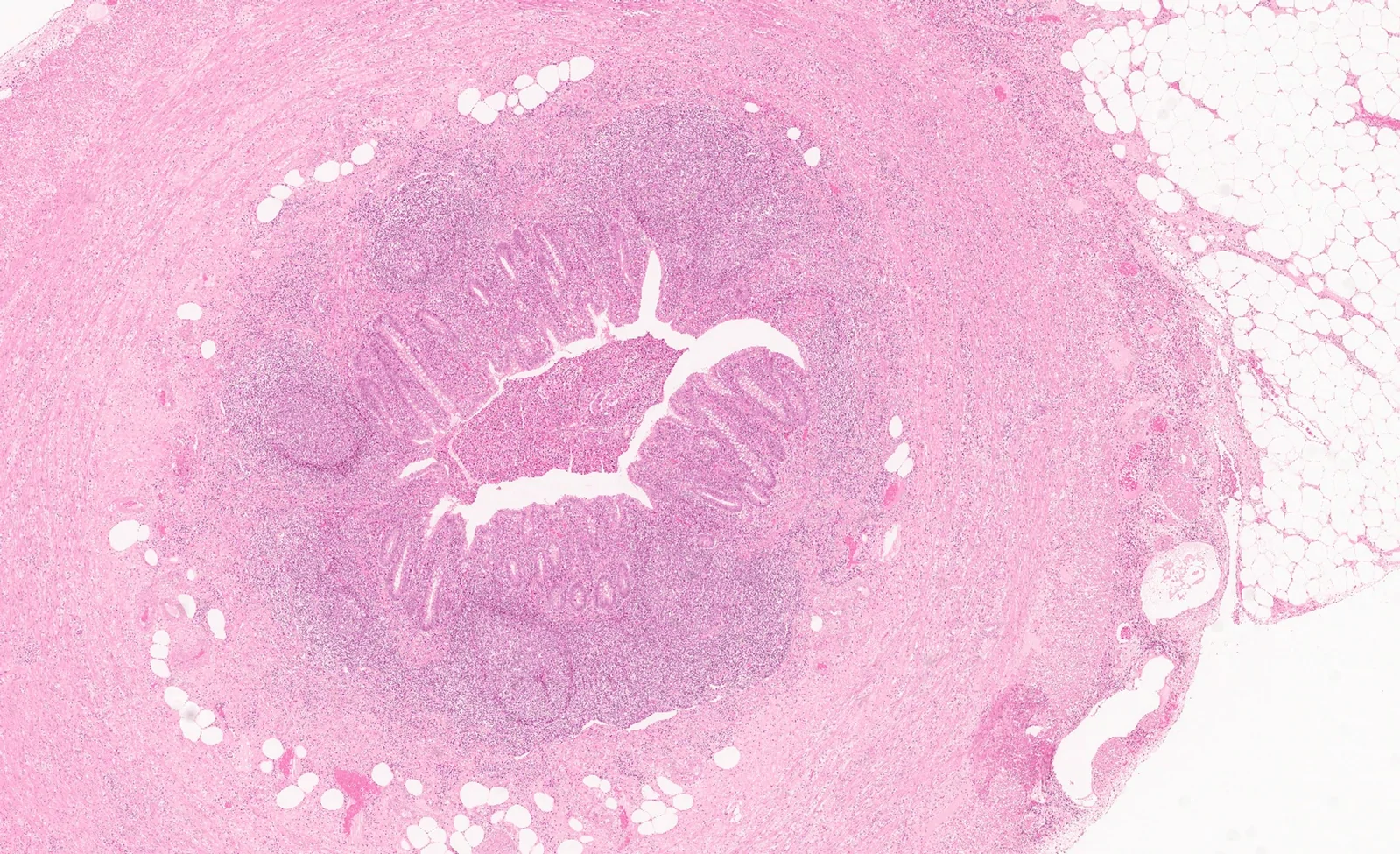
Dense neutrophilic infiltrate involving the appendiceal wall Transmural neutrophilic infiltration with follicular lymphoid hyperplasia and luminal exudate, consistent with acute phlegmonous appendicitis (H&E, original magnification x100)

**Figure 4:**
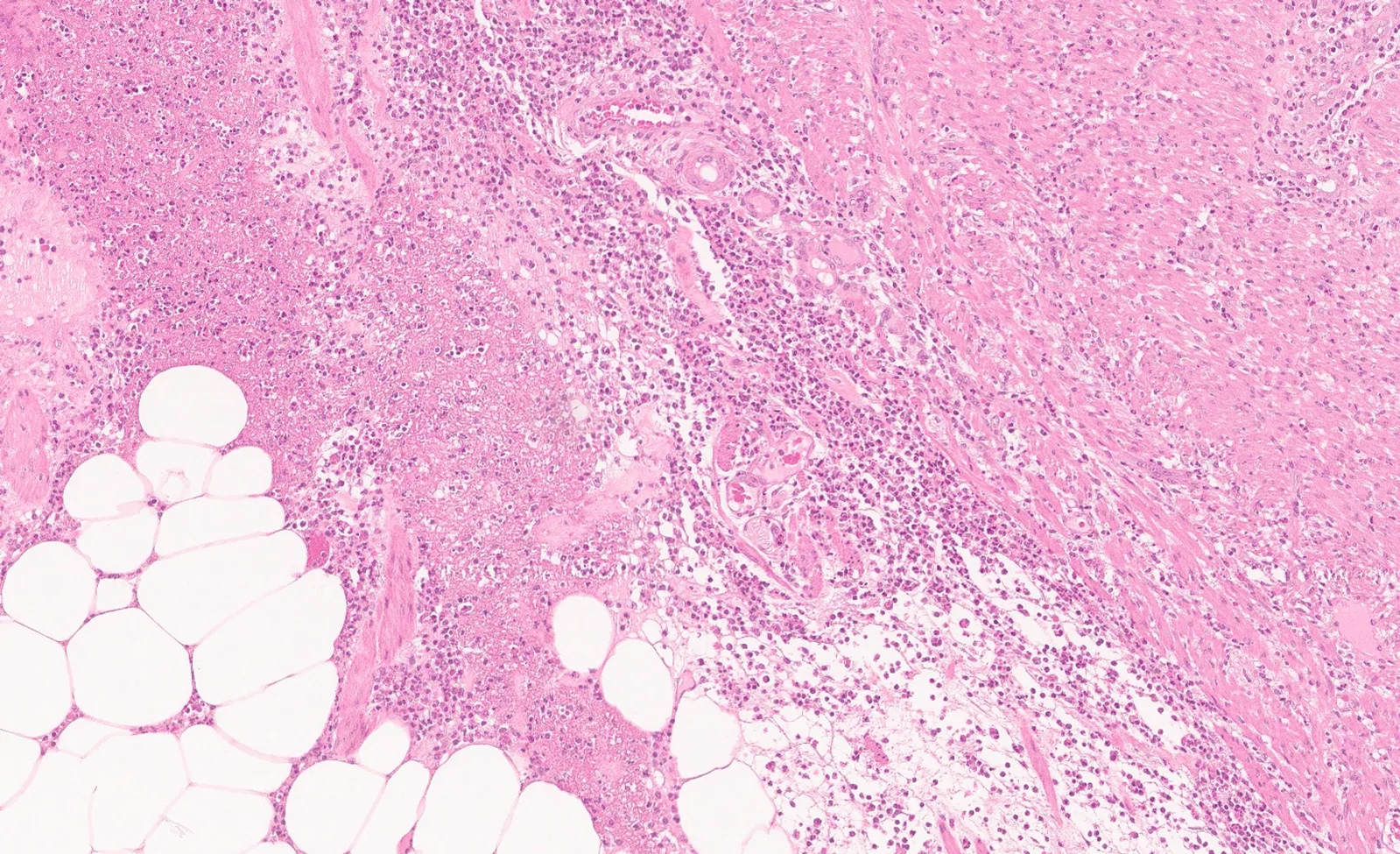
Focal serositis Acute inflammatory infiltrate with neutrophils and fibrin involving the serosa, extending into the periappendiceal adipose tissue, consistent with focal serositis (H&E, original magnification x200)

## Discussion

This case combines two unusual diagnostic circumstances: acute appendicitis involving a left-sided appendix in a patient with previously unsuspected intestinal malrotation and the initial suspicion of appendicitis arising from the incidental colonoscopic observation of purulent drainage from the appendiceal orifice.

Intestinal malrotation and left-sided appendicitis

Intestinal malrotation is a congenital abnormality resulting from incomplete or abnormal rotation and fixation of the midgut during embryological development. Although classically considered a pediatric condition, malrotation may remain undiagnosed into adulthood. Xiong et al. evaluated CT findings in 332 adults with intestinal malrotation and demonstrated considerable anatomical variability [2]. Of these patients, 188 were asymptomatic at diagnosis, while an ectopic cecum was identified in 40 patients, including a left-sided cecum in 29 cases [2]. These findings demonstrate the potential for malrotation to remain clinically silent into adulthood and the importance of CT in defining individual anatomy.

The relationship between congenital anatomical abnormalities and left-sided appendicitis has been described in the surgical literature. Akbulut et al. reviewed 95 published cases of left-sided acute appendicitis associated with congenital abnormalities, of which 66 were associated with situs inversus totalis and 23 with midgut malrotation [1]. 

Situs inversus and intestinal malrotation should be distinguished: situs inversus is characterized by mirror-image transposition of the viscera, whereas intestinal malrotation results from abnormal embryological rotation and fixation of the bowel. The present patient had intestinal malrotation without situs inversus.

Appendicitis associated with malrotation may present with left lower quadrant pain and mimic other conditions [3,4]. Ozkan et al., for example, described appendicitis associated with malrotation presenting clinically as left renal colic [4]. In older adults, acute diverticulitis represents another important diagnostic consideration.

CT is particularly valuable in this setting because it can simultaneously demonstrate appendiceal inflammation, characterize the position of the bowel and cecum, and identify features of the underlying rotational abnormality [2-5]. Preoperative recognition of this anatomy is also valuable for operative planning. In our patient, CT explained the apparent discrepancy between isolated left iliac fossa tenderness and an endoscopic finding strongly suggestive of appendicitis by demonstrating a predominantly left-sided colon and an inflamed appendix arising from the abnormally positioned cecum to the left of the midline.

Magnetic resonance enterography (MRE) may also represent a useful radiation-free imaging alternative for the evaluation of intestinal malrotation in selected non-emergency patients. MRE can demonstrate abnormal bowel positioning and assess the intestinal loops without ionizing radiation, although its role in adult intestinal malrotation remains less established than that of CT [6].

Colonoscopic identification of acute appendicitis

The second unusual feature of this case was the initial identification of appendiceal inflammation during elective colonoscopy. Although colonoscopy is not part of the routine diagnostic pathway for suspected acute appendicitis, appendicitis may occasionally be identified unexpectedly during colonoscopy performed for another indication.

Chang et al. retrospectively evaluated 21 patients in whom colonoscopy contributed to the diagnosis of appendicitis [7]. Appendicitis was considered confirmed in 20 patients. Colonoscopic abnormalities included surrounding mucosal edema in 19 patients, bulging of the appendiceal orifice in 18, hyperemia in 15, and purulent drainage from the appendiceal orifice in 7 [7]. These observations demonstrate that drainage of pus from the appendiceal orifice is an uncommon but recognized endoscopic manifestation of appendicitis.

Benatta reported the incidental colonoscopic identification of suppurative appendicitis, characterized by inflammatory changes at the appendiceal orifice and purulent discharge into the cecum [8]. Similarly, Idris et al. described an inflamed appendiceal orifice with projecting pus during elective colorectal cancer screening, prompting referral for surgical assessment [9].

Our case differs from these reports because the incidental colonoscopic finding occurred in the additional setting of previously undiagnosed intestinal malrotation and a left-sided appendix. The patient had experienced abdominal pain before the colonoscopy but attributed it to bowel preparation, while subsequent clinical examination demonstrated tenderness in the left rather than right iliac fossa. The colonoscopic finding therefore provided the initial specific clue to the presence of appendicitis despite an atypical clinical presentation.

These observations should not be interpreted as supporting colonoscopy as a routine diagnostic investigation for suspected acute appendicitis. Rather, characteristic inflammatory findings at the appendiceal orifice encountered incidentally during colonoscopy should prompt consideration of acute appendicitis and appropriate clinical and radiological evaluation.

Surgical considerations

Laparoscopic appendectomy is feasible in patients with intestinal malrotation, but recognition of the abnormal anatomy is important for operative planning. Conventional trocar placement is designed to provide triangulation toward a right-sided cecum and may therefore be suboptimal when the appendix is situated on the left.

Previous reports demonstrate that laparoscopic appendectomy can be successfully performed in patients with malrotation when port positioning is adapted to the individual anatomy [1,5,10]. Hassanat et al. recently described successful laparoscopic treatment of left-sided acute appendicitis in an adult with intestinal malrotation following preoperative CT characterization of the anatomy [10]. In our patient, right-sided working ports provided effective triangulation toward the left-sided appendix, allowing the procedure to be completed laparoscopically in approximately 45 minutes without intraoperative complications.

Overall, this case illustrates the importance of integrating clinical, endoscopic, and radiological findings when appendicitis presents in an atypical anatomical location. Recognition of the altered anatomy both clarified the left-sided presentation and facilitated planning of the laparoscopic approach.

## Conclusions

Acute appendicitis should remain within the differential diagnosis of left-sided abdominal pain when congenital abnormalities of intestinal rotation are present. Intestinal malrotation may remain unsuspected until adulthood and can substantially alter the expected location and clinical presentation of common abdominal diseases.

In this patient, the initial specific suspicion of appendicitis arose after purulent material was incidentally observed at the appendiceal orifice during elective colonoscopy. CT subsequently demonstrated previously undiagnosed intestinal malrotation and an inflamed appendix to the left of the midline. Recognition of the abnormal anatomy facilitated appropriate modification of the laparoscopic approach and successful appendectomy. Although colonoscopy has no routine role in the investigation of suspected acute appendicitis, purulent drainage from the appendiceal orifice is a recognized endoscopic manifestation of appendiceal inflammation. This case highlights the value of integrating clinical, endoscopic, and radiological findings when a common surgical emergency presents in an uncommon anatomical location.
